# Supplementary figures and images for: Differential dysregulation of β-TrCP1 and -2 by HIV-1 Vpu leads to inhibition of canonical and non-canonical NF-κB pathways in infected cells
Source: mBio. 2023 Jun 21;14(4):e03293-22. doi: 10.1128/mbio.03293-22 (PMC10470808; doi:10.1128/mbio.03293-22)

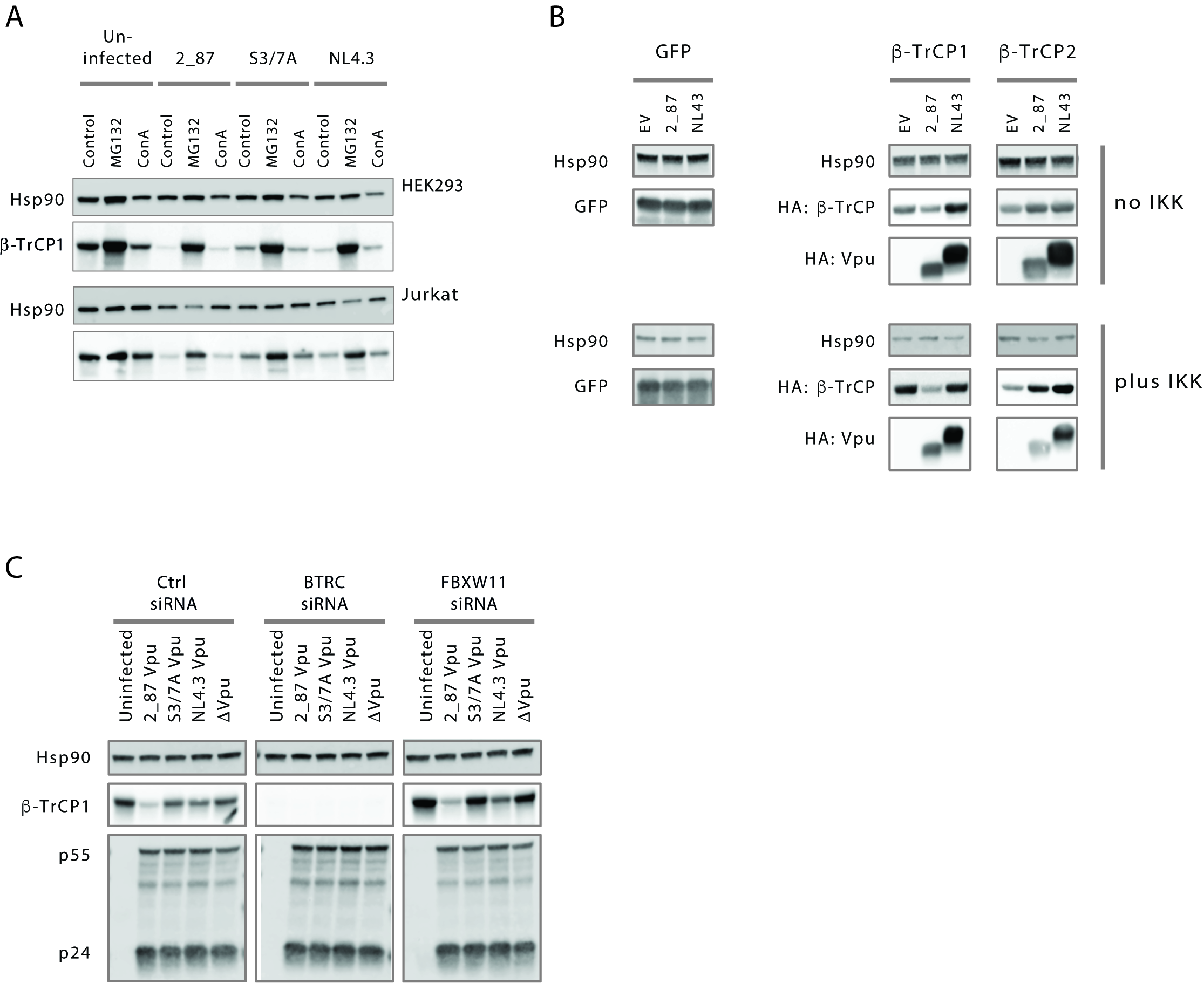

Supplement: Figure S1 — (A) HEK293T and Jurkat cells were infected with recombinant NL4.3 proviruses engineered to express either highly active 2_87 Vpu, 2_87 S3/7A Vpu, NL4.3 Vpu or no Vpu (Δ Vpu) at an MOI of 5, and treated with proteasomal inhibitor MG132 (10μM) or concanamycin A (50 nM) for 6 h prior to harvest at 48 h. Cell lysates were analysed by western blot for Hsp90 (loading control) and endogenous β-TrCP1 levels. (B) HEK293T cells were co-transfected with GFP or HA-tagged β-TrCP1 or -2 plus Vpu or empty vector control, in the presence (+IKK) or absence (no IKK; empty vector) of active signaling. Twenty-four hours after transfection, cell lysates were harvested and analysed by western blot for GFP, HA (Vpu and β-TrCP) and Hsp90 (loading control). (C) HEK293T cells were pre-treated with siRNA for β-TrCP1 (BTRC), -2 (FBXW11) or control before infecting with recombinant NL4.3 proviruses engineered to express either highly active 2_87 Vpu, 2_87 S3/7A Vpu, NL4.3 Vpu or no Vpu (Δ Vpu) at an MOI of 4.Forty-eight hours after infection cells were lysed and analyzed by western blot for Hsp90 (loading control), endogenous β-TrCP1 levels and HIV-1 Gag (major bands show p55 and p24). [file mbio.03293-22-s0001.tif]

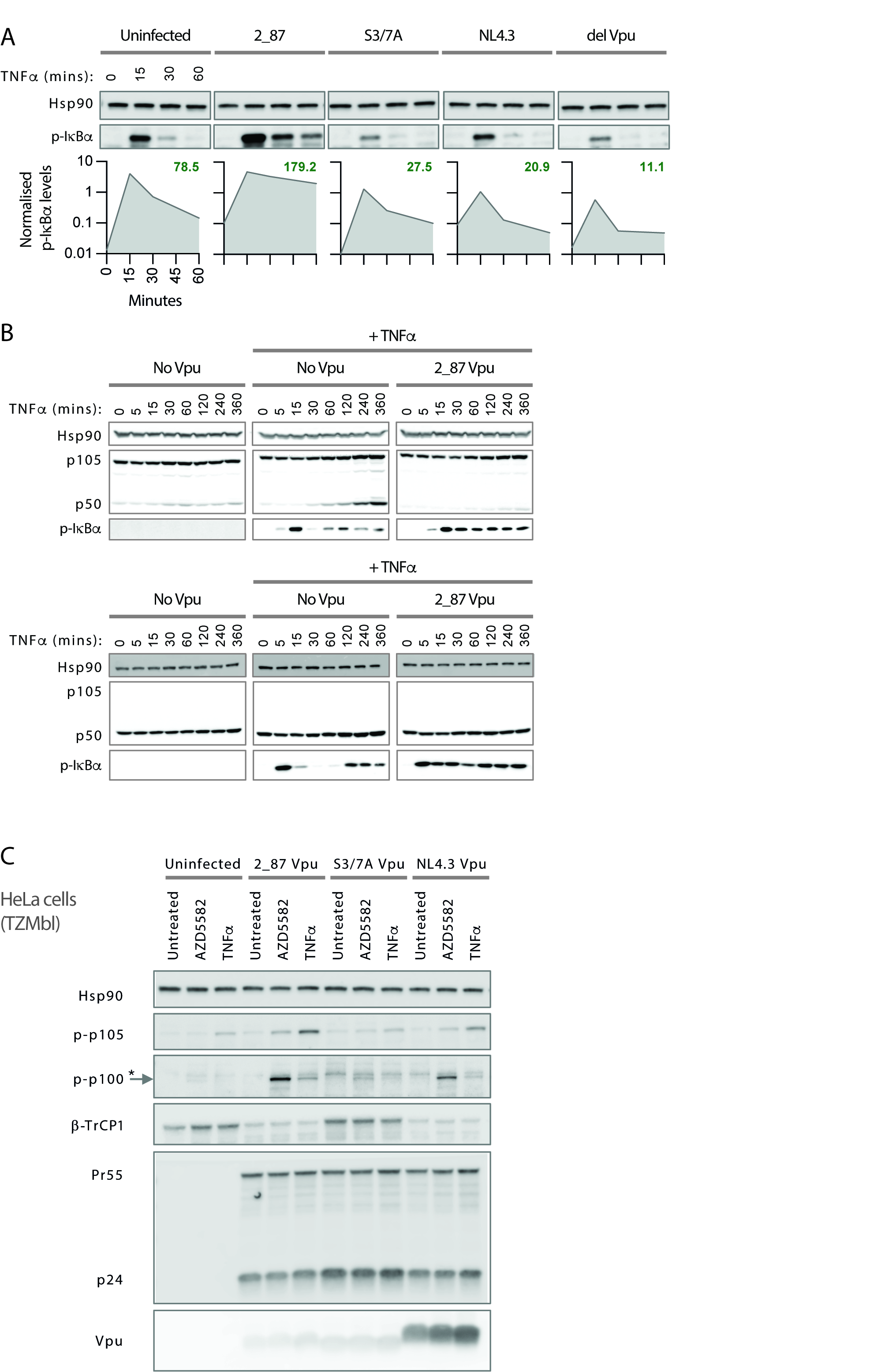

Supplement: Figure S2 — (A) IκBα stabilization timecourse in Jurkat cells. CD4+ Jurkat T cells were infected with viruses expressing either 2_87, 2_87 S3/7A, NL4.3 or no Vpu (Δ Vpu) at an MOI of 3. Forty-eight hours after infection, cells were treated with 10 ng/ml TNFα, and time points were harvested at 0, 15, 30, and 60 minutes following treatment. Samples were analysed by western blot for Hsp90 (loading control) and phospho-IκBα. Band intensities for p-IκBα are shown below each blot, normalized to Hsp90 for each sample and to positive controls for p-IκBα, as appropriate, per blot (not shown in the image). Numbers shown in bold green text on each graph represent the calculated area under the curve (AUC). (B) p105 processing timecourse with TNFα. Transient p105 processing assays were performed by co-transfecting HA-p105 (top panel) or HA-p50 (bottom panel) with 2_87 Vpu or empty vector control. Eighteen hours after transfection, cells were stimulated with 10 ng/ml TNFα, or mock treated, and time points harvested for western blotting at 0, 5, 15, 30, 60, 120, 240, and 360 minutes. Blots were probed for HA (p105 and 50 in the top panel; p50 only in the bottom panel) and Hsp90 (loading control). Phospho-IκBα blots were included as positive controls for NF-κB signal activation. (C) Recombinant NL4.3 proviruses engineered to express either 2_87, 2_87 S3/7A or NL4.3 Vpu were used to infect HeLa TZMbl cells at an MOI of 3. Forty-two hours after infection, cells were treated with 200 nM AZD5582 or 10 ng/ml TNFα, or left untreated. Six hours after treatment, cells were harvested and western blotted for Hsp90 (loading control), phospho-p105 (Ser932), phospho-p100 (Ser866/870) and β-TrCP1. * denotes non-specific band. HIV-1 Gag (p55 and p24) and Vpu were blotted as controls for infection levels. [file mbio.03293-22-s0002.tif]
