# Supplementary material for: Differential dysregulation of β-TrCP1 and -2 by HIV-1 Vpu leads to inhibition of canonical and non-canonical NF-κB pathways in infected cells
Source: mBio. 2023 Jun 21;14(4):e03293-22. doi: 10.1128/mbio.03293-22 (PMC10470808; doi:10.1128/mbio.03293-22)
Supplement: TABLE S1 — List of primers used for generating recombinant plasmids and mutant constructs. [file mbio.03293-22-s0003.docx]

**Supplementary Table 1**

List of primers used for generating recombinant plasmids and mutant constructs

| **Name** | **Sequence (5’-3’)** |
| --- | --- |
| **Vpu mutagenesis primers (for codon-optimised sequences)** | |
| NL4.3 CO S52A F | GAGCGCGCCGAGGACGCCGGCAACGAG |
| NL4.3 CO S56A F | GGCAACGAGGCCGAGGGCGAGGTGAGCG |
| 2_87 CO S53A F | GAGCGCGCCGAGGACGCCGGCAACGAG |
| 2_87 CO S53E F | GAGCGCGCCGAGGACGAGGGCAACGAG |
| 2_87 CO S57A F | GGCAACGAGGCCGAGGGCGACCAGGAGG |
| 2_87 CO S57E F | GGCAACGAGGAGGAGGGCGACCAGGAGG |
| 2_87 CO S65A F | AGGAGGAGCTGGCCGCCCTGGTGGA |
| 2_87 CO R45K F | CCGCCTGATCGACAAGATCCGCGAGCGC |
| 2_87 CO A50V F | ATCCGCGAGCGCGTGGAGGACAGCG |
| 2_87 CO G59R F | ACGAGAGCGAGCGCGACCAGGAGG |
| 2_87 CO E62G F | AGGGCGACCAGGGGGAGCTGAGCG |
| 2_87 CO SS62/63AA F | AGGGCGACCAGGCCGCCCTGAGCGCCCT |
|  |  |
| **Primers for insertion of 2_87 Vpu into HIV-1 NL4.3 IRES-eGFP** | |
| 2_87 SnaBI F | GCGCATTACGTAATGAAATCTTTAGAGACATTAGC |
| 2_87 XbaI R | CGCGATTCTAGAGATCATCAATATCCCAAGG |
| SnaBI reversion 2_87 F (J037) | CCTTGGGATATTGATGATCTGTAGTGCTACAGAAAAATTGTGGG |
| 2_87 S53,57A F | TGATAGAATAAGAGAAAGAGCAGAAGACGCTGGCAATGAGGCTGAAGGGGATCAGGAAGAATTATCAG |
|  |  |
| **NF-κB primers** | |
| Human BTRC F | ATGGACCCGGCCGAGG |
| Human BTRC XhoI F | ATTCCTCGAGATGGACCCGGCCGAGG |
| Human BTRC R | TTATCTGGAGATGTAGGTGTATGTTCG |
| Human BTRC NotI R | ATTAGCGGCCGCTTATCTGGAGATGTAGGTGTATGTTCG |
| Human IKKbeta EcoRI kzc F | GCTAGAATTCGCCACCATGAGCTGGTCACCTTCCCTG |
| Human IKKbeta R | TCATGAGGCCTGCTCCAGG |
| IKKbeta SS177,181EE F | AAGGAGCTGGATCAGGGCGAACTTTGCACAGAATTCGTGGGGACC |
| Human p105 F | ATGGCAGAAGATGATCCATATTTGG |
| Human p105 XhoI kzc F | GCGCCTCGAGGCCACCATGGCAGAAGATGATCCATATTTGG |
| Human p105 R | CTAAATTTTGCCTTCTAGAGGTCC |
| Human p105 NotI R | GCGCGCGGCCGCCTAAATTTTGCCTTCTAGAGGTCC |
| Human p50 stop NotI R | GCGCGCGGCCGCCTATCCATGCTTCATCCCAGCATTAG |
| Human p100 F | ATGGAGAGTTGCTACAACCCA |
| Human p100 EcoRI kzc F | GCGCGAATTCGCCACCATGGAGAGTTGCTACAACCCA |
| Human p100 R | TCAGTGCACCTGAGGCTGG |
| Human p100 NotI R | GCGCGCGGCCGCTCAGTGCACCTGAGGCTGG |
| Human NIK F | ATGGCAGTGATGGAAATGGCCTG |
| Human NIK EcoRI kzc F | GCGCGAATTCGCCACCATGGCAGTGATGGAAATGGCCTG |
| Human NIK R | TTAGGGCCTGTTCTCCAGCTGG |
| Human NIK XhoI R | GCGCCTCGAGTTAGGGCCTGTTCTCCAGCTGG |
